# Effective Infodemic Management: A Substantive Article of the Pandemic Accord

**DOI:** 10.2196/51760

**Published:** 2023-09-20

**Authors:** Kazuho Taguchi, Precious Matsoso, Roland Driece, Tovar da Silva Nunes, Ahmed Soliman, Viroj Tangcharoensathien

**Affiliations:** 1 Permanent Mission of Japan to the International Organizations in Geneva Geneva Switzerland; 2 University of the Witwatersrand Johannesburg South Africa; 3 Ministry of Health, Welfare and Sports The Hague Netherlands; 4 Mission Permanente du Brésil auprès de l'Office des Nations Unies Geneva Switzerland; 5 Permanent Mission of the Arab Republic of Egypt to the United Nations Office and Other International Organizations Geneva Switzerland; 6 International Health Policy Program Ministry of Public Health Nonthaburi Thailand

**Keywords:** Pandemic Accord, infodemic, infodemic management, COVID-19, social media, Intergovernmental Negotiating Body, INB, INB Bureau, World Health Organization, WHO, misinformation, disinformation, public health

## Abstract

Social media has proven to be valuable for disseminating public health information during pandemics. However, the circulation of misinformation through social media during public health emergencies, such as the SARS (severe acute respiratory syndrome), Ebola, and COVID-19 pandemics, has seriously hampered effective responses, leading to negative consequences. Intentionally misleading and deceptive fake news aims to harm organizations and individuals. To effectively respond to misinformation, governments should strengthen the management of an “infodemic,” which involves monitoring the impact of infodemics through social listening, detecting signals of infodemic spread, mitigating the harmful effects of infodemics, and strengthening the resilience of individuals and communities. The global spread of misinformation requires multisectoral collaboration, such as researchers identifying leading sources of misinformation and superspreaders, media agencies identifying and debunking misinformation, technology platforms reducing the distribution of false or misleading posts and guiding users to health information from credible sources, and governments disseminating clear public health information in partnership with trusted messengers. Additionally, fact-checking has room for improvement through the use of automated checks. Collaboration between governments and fact-checking agencies should also be strengthened via effective and timely debunking mechanisms. Though the Intergovernmental Negotiating Body (INB) has yet to define the term “infodemic,” Article 18 of the INB Bureau’s text, developed for the Pandemic Accord, encompasses a range of actions aimed at enhancing infodemic management. The INB Bureau continues to facilitate evidence-informed discussion for an implementable article on infodemic management.

While social media has proven valuable for public health officials to disseminate crucial information during the COVID-19 pandemic, it has also been misused by many internet users to spread misinformation and fake news. This has hindered the public health response, particularly concerning issues like antivaccine and antimask sentiments. Fake news, which is intentionally misleading and deceptive, aims to harm organizations and individuals [1].

Misinformation is commonly shared on platforms such as Twitter, YouTube, Facebook, messaging applications, and personal websites. This has exacerbated the severity of the pandemic, eroded trust in public health experts, and led to nonadherence to public health and social measures [2,3].

Health-related misinformation on social media ranges from 0.2% to 28.8% [4]. Twitter, Facebook, YouTube, and Instagram play a critical role in rapidly spreading far-reaching misinformation. The increase in unreliable health information has delayed care provision and contributed to the dissemination of hateful and divisive rhetoric. However, social media can also be a useful tool to combat misinformation during crises [4].

Misinformation during large-scale infectious disease outbreaks has been observed since 2000, for example, during the SARS (severe acute respiratory syndrome) and Ebola outbreaks. False information is often linked to prevention, treatment, risk factors, transmission modes, and complications, and propagated by vaccine conspiracy theories [5]. A content analysis of 6600 randomly selected English-language tweets revealed that 22% contained false or partially false information, with the political nature of vaccines being most prevalent [6].

The World Health Organization (WHO) proposes addressing the challenge of managing an “infodemic” effectively using these five interconnected areas: (1) closely monitoring and measuring the impact of infodemics during health emergencies through social listening [7]; (2) detecting signals and understanding the spread and risk of infodemics; (3) responding with interventions that mitigate and protect against the harmful effects of infodemics; (4) assessing infodemic interventions and strengthening the resilience of individuals and communities; and (5) promoting the development, adaptation, and application of interventions and toolkits for infodemic management [8].

Messages should be based on scientific evidence and actionable behavioral change. They should be tailored to different population groups to enable them to make informed decisions to protect themselves and their communities during a pandemic. Vulnerable groups, such as women and youth, are particularly susceptible to misinformation. Health authorities should engage in multisectoral, interdisciplinary, and multilevel collaborations, including with the private sector, especially social media corporations and influencers [9]. Integrating artificial intelligence into social listening can provide real-time information about public discourse and support informed infodemic responses [10].

Digital health literacy refers to the ability to critically analyze elements presented in social media and determine their accuracy or credibility. Strengthening digital health literacy in the population can be done through education and engagement with communities, health professionals, and decision makers. A study revealed that less than half of health professionals possess a high level of digital health literacy [11]. In assessing digital health literacy in the population, a scoping review showed that the most commonly used tool is the eHealth Literacy Scale [12]. This short questionnaire consists of 8 questions with a 5-point Likert scale [13]. The eHealth Literacy Scale can be modified and used to regularly monitor and strengthen digital health literacy in the population.

Effective response to misinformation necessitates multisectoral and multidisciplinary collaboration. Researchers can identify leading sources of misinformation, including superspreaders. Media organizations can identify and debunk misinformation. Technology platforms can monitor and address misinformation by reducing the distribution of false or misleading posts and guiding users to health information from credible sources. Governments can disseminate clear public health information in partnership with trusted messengers [14].

Fact-checking has room for improvement. One study indicated that disinformation incorporates features from both propaganda and mainstream news, making it more challenging to detect. The scientific community should develop automated fact-checking processes that consider the degree of veracity [15]. Countries can apply Factcheck, a powerful tool in the fight against misinformation [16], along with effective and timely debunking mechanisms.

In response to the COVID-19 pandemic, WHO member states decided to draft and negotiate a WHO convention, agreement, or other international instrument on pandemic prevention, preparedness, and response (referred to as “WHO CA+”). The goal is to adopt this instrument under Article 19 or other provisions of the WHO Constitution as deemed appropriate by the Intergovernmental Negotiating Body (INB). This decision was mandated during the World Health Assembly Special Session in December 2021 [17], with the envisaged adoption of WHO CA+ by the 77th session of the World Health Assembly in May 2024.

Infodemics is an emerging challenge brought about by the widespread use of social media. This is the first time in history that the INB is discussing how to address misinformation. Article 18 of the INB Bureau’s text [18], “Communication and Public Awareness,” encompasses a range of actions on public communication that use scientific knowledge to increase awareness through community engagement and health literacy. Infodemic management is crucial to combat false or misleading information and disinformation. This includes regular social listening to assess the prevalence and profiles of misinformation. Active participation of the INB in the discussion will lead to an implementable article. We, the members of the INB Bureau [19], will continue to facilitate evidence-informed negotiations toward effective infodemic management.

## Abbreviations

INB: Intergovernmental Negotiating Body
SARS: severe acute respiratory syndrome
WHO: World Health Organization
